# A new removable airway stent

**DOI:** 10.3402/ecrj.v3.30010

**Published:** 2016-09-06

**Authors:** Tore Amundsen, Sveinung Sørhaug, Håkon Olav Leira, Stig Sverre Tyvold, Thomas Langø, Tommy Hammer, Frode Manstad-Hulaas, Erney Mattsson

**Affiliations:** 1 Department of Thoracic Medicine, St. Olavs Hospital, Trondheim, Norway; 2 Faculty of Medicine, Institute of Circulation and Medical Imaging, Norwegian University of Technology and Science (NTNU), Trondheim, Norway; 3 Aleris Hospital, Trondheim, Norway; 4 Department of Medical Technology, SINTEF, Trondheim, Norway; 5 Norwegian National Advisory Unit for Ultrasound and image-guided therapy, St Olavs Hospital, Trondheim, Norway; 6 Department of Radiology, St, Olavs Hospital, Trondheim, Norway; 7 Department of Vascular Surgery, St. Olavs Hospital, Trondheim, Norway

**Keywords:** interventional bronchoscopy, airway stent, malignant airway obstruction, SEMS, lung cancer

## Abstract

**Background:**

Malignant airway obstruction is a feared complication and will most probably occur more frequently in the future because of increasing cancer incidence and increased life expectancy in cancer patients. Minimal invasive treatment using airway stents represents a meaningful and life-saving palliation. We present a new removable airway stent for improved individualised treatment.

**Methods:**

To our knowledge, the new airway stent is the world's first knitted and uncovered self-expanding metal stent, which can unravel and be completely removed. In an *in vivo* model using two anaesthetised and spontaneously breathing pigs, we deployed and subsequently removed the stents by unravelling the device. The procedures were executed by flexible bronchoscopy in an acute and a chronic setting – a ‘proof-of-principle’ study.

**Results:**

The new stent was easily and accurately deployed in the central airways, and it remained fixed in its original position. It was easy to unravel and completely remove from the airways without clinically significant complications. During the presence of the stent in the chronic study, granulation tissue was induced. This tissue disappeared spontaneously with the removal.

**Conclusions:**

The new removable stent functioned according to its purpose and unravelled easily, and it was completely removed without significant technical or medical complications. Induced granulation tissue disappeared spontaneously. Further studies on animals and humans are needed to define its optimal indications and future use.

Benign and malignant airway obstruction (MAO) may affect individuals at all ages. The physicians must think and act differently in each case, depending on the cause, the characteristics of the airway obstruction, and the individual patient. The treatment options are numerous, including interventional bronchoscopy, systemic treatment, and endobronchial and extrathoracic irradiation. The need for optimal individualised treatment is a fact (1–3).

Benign airway obstruction comprises fibrous membrane stenosis, post-infectious granulomas and strictures, tracheobronchomalacia, foreign bodies, and severe haemoptysis. MAO is more frequent in the adult population, expressed either as intrabronchial tumour growth or external tumour compression, not seldom complicated with trachea-esophageal fistulas and airway stenosis (4). Figure 1 shows an example of an MAO before and after multimodal treatment. MAO will most probably occur more frequently in the future because of increasing cancer incidence and increased life expectancy. Patients with MAO are often suffering from mild to severe dyspnoea, cough, recurrent pneumonia and frequent hospitalisation, and the prognosis varies considerably (5). These secondary symptoms can also become life-threatening. Minimal invasive treatment using airway stents often represents a meaningful and life-saving palliation, with or without local or systemic supplementary therapy (6–13) (Fig. 1).

**Fig. 1:**
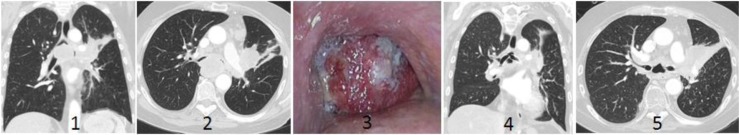
Illustration of a life-threatening malignant central airway obstruction. CT images, 1–2: coronal and transversal slices showing almost complete occlusion of the right and left main bronchus by a tumour; video bronchoscopy, image 3: protruding tumour at the level of carina; CT images, 4–5: coronal and transversal slices showing the reestablished lumen of both main bronchi after stent implantation and radiotherapy.

Thermal ablation (e.g. laser, diathermia and cryo techniques) or brachytherapy applied with bronchoscopy is often used to treat MAO. Thermal ablation is contraindicated in stenosis caused by pure external tumour compression, often leaving stent therapy as the best choice. There are different types of stents available on the market. Some silicone stents are radiopaque and some are not; deployment needs rigid bronchoscopy and general anaesthesia, but these stents can be removed. Self-expanding metal stents (SEMS) are visible by X-ray; deployment can be performed with flexible bronchoscopy using conscious sedation combined with local anaesthetics. Uncovered SEMS can be difficult to remove because of epithelisation, ingrowth of tumour and granulomatous tissue. This drawback is less pronounced in first weeks after deployment and less a problem in covered stents. Uncovered SEMS are avoided in benign airway disorders and in patients with MAO who have a longer life expectancy. Complications to airway stents are migration within the airways, granuloma formation, mucostasis and pneumonia, mucosal ischaemia and fistulas. This is because of the radial pressure on neighbouring tissue induced by the stent. Complications may also be associated with the underlying condition or comorbidity (12, 14, 15). Correct choice of stent material, covered or not covered, shape, size and radial strength reduce the complication rate. Long-lasting stents may need subsequent dilatation (4, 12, 16).

Lacking the possibility of removing uncovered SEMS, especially after 6–8 weeks, is a clinically important limitation of the functionality of the stent. Covered SEMS are often used in patients with MAO and short lifetime expectancy (1, 3, 6, 17–19). First, removable uncovered SEMS, which do not obstruct airway branches and thereby maintain ventilation, could be a good alternative for patients with MAO who have a longer life expectancy and for those with benign airway disorders. Second, temporary stenting would be favourable in many cases, such as during and after interventional bronchoscopy or following extrathoracic radiation and brachytherapy to reduce the development of phthisis, scar and stenosis formation (20). Third, the physician would appreciate the opportunity to remove the stent at any time, also during the deployment to guarantee the final stent placement to be optimal (21). In this study, we performed both acute and chronic observations in an animal model with spontaneous breathing pigs. The aim was to test the basic functionality of a new removable knitted stent that can unravel and be completely removed, a ‘proof-of-principle’ study.

## Methods

### Removable stent

**Fig. 2:**
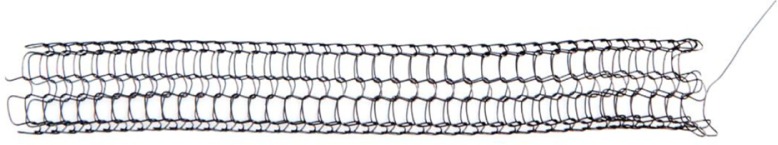
The new removable stent (magnified) made by self-expanding nitinol with a thread that extends beyond the tubular part. The end of the thread can be placed at a distance and be used to unravel the device to the thread from which it was made and remove the stent.

The removable stent used in the two current experiments represents a new type of uncovered self-expanding metal stent made by the memory alloy nitinol (Fig. 2). The stent is knitted and can thereby unravel into the single thread from which it was made. Furthermore, the knitting technique makes it possible to produce the stent for a variety of indications, varying thread thickness and mesh size giving varying radial strengths, dimensions (length and diameter) and shapes, including Y-formation. It can therefore be customised and made to suit different tumour and patient characteristics. Therefore, it may respond to the clinical needs for individualised treatment, and in that case, it can be delivered within 24 h. The usual situations should, however, be possible to cover by devices from the shelf in the hospitals doing the procedures. In this study, the stents were knitted with a nitinol thread having a diameter of 0.1 mm. The knitting pattern was adjusted to reach a radial force corresponding to the commercially available stents of today, such as the Ultraflex stent. The stents were cooled down in wet ice before being packed into delivery sheaths, which were adjusted from standard vascular introducers with an outer diameter of 6 French. The delivery sheaths containing the stents were soaked in ethanol as disinfection and thereafter sterilised.

The stent is knitted from a nitinol thread. Nitinol is a memory alloy, and the stent thereby belongs to the group of SEMS. There is usually no need for any balloon dilatation after deployment of the stent. The mechanical impact and radial force can be predefined and specified according to the individual needs, which increases the possibilities for successful individualised treatment. Before this study, the new removable stent has been thoroughly tested, *in vitro* for its mechanical properties and as vascular stent in iliac arteries in sheep in acute and chronic studies, with successful unravelling. The stent is ordered and produced online based on specifications of needs for a specific person and condition. It is produced at GraftCraft AB, Borås, Sweden. The stent is not approved for medical use in humans, but based on this and further studies, this is the plan.

### Aim

**Fig. 3:**
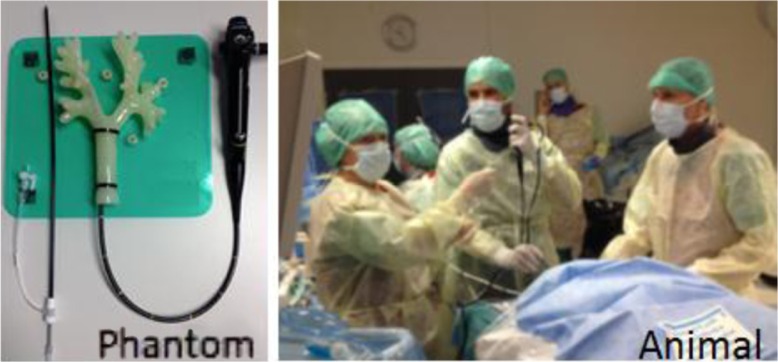
The phantom model where the stent, introducer system and removability were tested before the animal study which took place at the Operating Room of the Future, ORF.

The aim of the study was to test whether the new removable stent would function according to its purpose, in the airways, in terms of efficacy (deployment and accurate placement), safety (adverse events) and the ability to be removed (unravelling characteristics). The study was performed in an acute and a chronic setting using an *in vivo* animal study with two spontaneously breathing pigs – ‘A proof-of-principle study’. The procedure was first tested in a phantom model and subsequently in the animal model (Fig. 3).

### Animal model – spontaneously breathing pig

We established a novel *in vivo* animal model with spontaneous breathing anaesthetised pigs. The pigs were acclimatised for 1 week before the studies in the animal research department and fasted overnight before each experiment. Subcutaneous premedication was given (azaperon 16 mg/kg body weight (mg/kg bw), diazepam 0.25 mg/kg bw, ketamine 3 mg/kg bw and 1 mg atropinsulphate). Anaesthesia was given through a venous catheter in a marginal ear vein. Induction consisted of ketamine 3–4 mg/kg bw and sodium pentothal 2–3 mg/kg bw. The pig received a temporary or orotracheal tube for transportation between buildings. Maintenance anaesthesia was given as a continuous intravenous infusion of ketamine, midazolam and fentanyl.

The orotracheal tube was removed right before the start of the stenting procedure. To achieve spontaneous breathing and open airways, the jaws and tongue were fixed with smooth textile bonds. Adequate oxygenation (oxygen saturation 96–100%) was maintained by 10 l/min O_2_ supply on a nasal tube with an inner diameter of 4.0 mm. Complementary ketamine, midazolam and fentanyl were given when the respiratory rate increased, respiration deepened and/or the eyelash reflex was elicited by finger stroke. Heart rate was kept between 60 and 100 beats/min and blood pressure was kept within 100–120/60–80 mmHg.

### Flexible bronchoscopy and stenting procedure

We used a flexible bronchoscope (Olympus BF 1T240, Olympus, Tokyo, Japan, 5.3 mm, 2.0 mm work channel) for inspection, deployment, unravelling and removal of the stents. The operating theatre was located at the Operating Room of the Future (ORF), which is dedicated to research and development activities. Deployment was facilitated by using a guide wire and an in-house-built introducer. The deployment and removal procedure was documented and guided through bronchoscopy videos and images, X-ray (fluoroscopy) and cone beam CT acquisition.

### Acute study (Pig 1)

Deployment and accurate placement, subsequent unravelling and complete removal of differently sized stents in differently sized airways in both lungs were performed with a flexible bronchoscope (Table 1). During the unravelling procedure, the forceps that grabbed the extended thread were operated through a catheter to achieve a counter pressure on the tubular part of the stent, ensuring stable stent position during unravelling. The catheter with the forceps was introduced side by side and fixed to the tip of the bronchoscope with an elastic band. The animal was observed 8 h between placement and removal of the stent.

**Table 1:** Study design

Acute study D1 (Pig 1)	Chronic study D1 (Pig 2)	Chronic study D14	Chronic study D18
Right MB: 14×20 mm stent, placement without GW, stent unravelling and removal	Right distal MB (to lower lobe): 8×20 mm stent, placement with GW	Bronchoscopy, mucus removal, unravelling and complete removal of the proximal stent	Bronchoscopy, removal of airway, including distal stent
Left MB: 11×20 mm stent, placement with GW, stent unravelling and removal	Right proximal MB: 14×20 mm stent, placement with GW		

D – Day, MB – main bronchus, GW – guide wire.

### Chronic study (Pig 2)

Day 1: Deployment and accurate placement of stents, leaving one peripheral (8×20 mm) and one more proximal stent (11×20 mm) in the right (main) bronchus (Table 1). Thereafter, the animal was transported to the Animal Care Facility Unit (ACF) at Norwegian University of Technology and Science (NTNU) to recover from anaesthesia and was fed, stalled and observed for 14 days.

Day 14: Bronchoscopy during which the operator grabbed the proximal end of the thread with the forceps, pulled carefully and unravelled the proximal stent as one single thread in one sequence, as described above in the acute study. The distal stent was left behind. The animal was observed in the ACF for another 4 days.

Day 18: Bronchoscopy was performed to study the conditions and healing of the proximal right main bronchus after removal of the proximal stent and control of the position and bronchial condition of the residual distal stent.

In the acute and at all stages of the chronic study, acquisition of bronchoscopy images and videos were collected, together with X-ray (fluoroscopy) and cone beam CT images. During anaesthesia, the pig showed no spontaneous movement, but showed normal cardio-pulmonary functions and breathed spontaneously during the whole experiment.

### Ethical aspects

The Local Ethics Committee of the Norwegian Experimental Animal Board approved the experiments in accordance with the Helsinki Convention for the use and care of animals.

## Results

### Acute study (Pig 1)

**Fig. 4:**
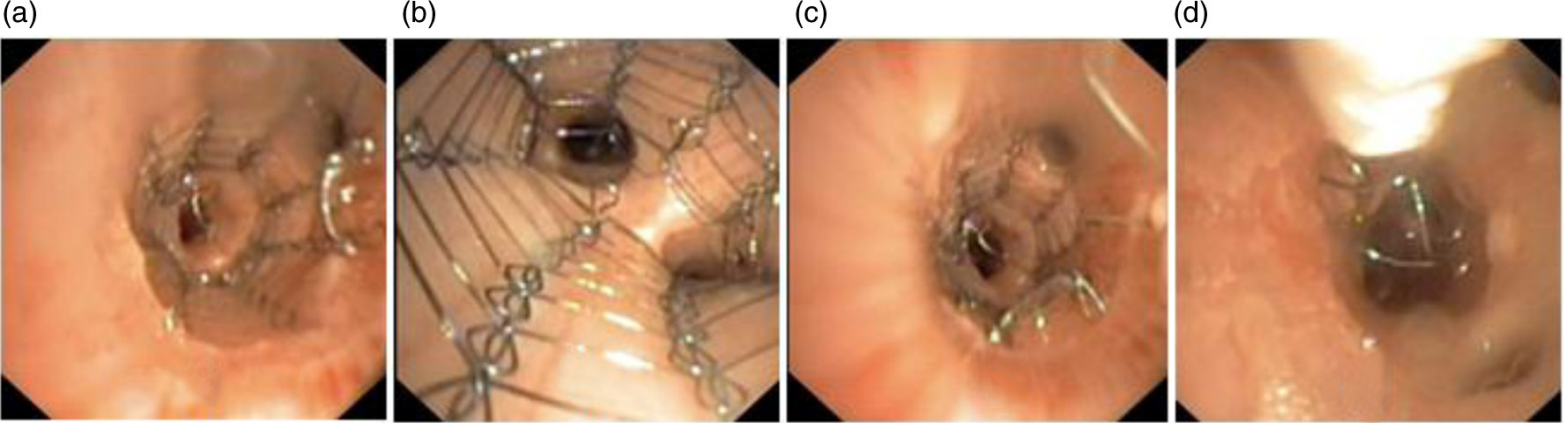
Acute study: Day 1 – placement and unravelling phase – placement of the stent, a (distant view) and b (close view). The unravelling procedure, midways and before complete removal, c and d, respectively.

The two stents (size 11×20 mm and 14×20 mm) were successfully deployed and accurately placed in the central airways, using a flexible bronchoscope and an in-house-built introducer and guide wire. The stents remained in their fixed positions before they were successfully unravelled and completely removed from the airways, carefully, in one sequence as a single thread (Fig. 4). The stents did not penetrate into or get stuck in the bronchial wall. The video bronchoscopy revealed no harm to the bronchial wall, no bleeding and no other complications, including clinical significant mucus retention. The stent, introducer, technique and procedure functioned according to its purpose. The results were found sufficiently successful in terms of efficacy and safety to proceed to the chronic part of the study.

### Chronic study (Pig 2)

Day 1: The two stents were successfully deployed and accurately placed in the right bronchial tree, one in distal position, lobe bronchus (8×20 mm stent), and the other in a more proximal position, main bronchus (11×20 mm stent), using the same instruments and technique as described in the Acute study section.

**Fig. 5:**
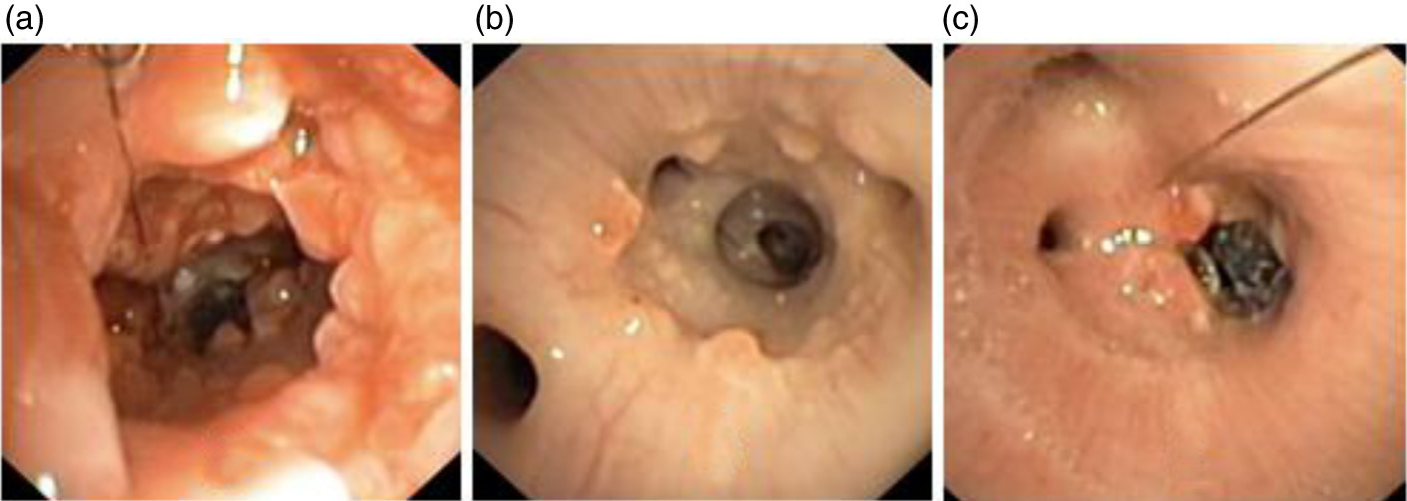
Chronic study: (a) Day 14 – after removal of proximal stent, and polyps are seen, (b) Day 18 – same area 4 days later showing dramatic healing and (c) Day 18 – view of the residual distal stent.

Day 14: The extended thread was immediately found using the bronchoscope, it was grasped with a forceps and the stent was easy to unravel and completely remove from the airways, with the technique described in the Methods section. The stent was carefully pulled out as a single thread in one sequence. The procedure was effective and safe, and was performed without technical or medical complications (Fig. 5). The video bronchoscopy revealed no harm to the bronchial wall, clinically significant mucus retention or other complications. A superficial minimal bleeding was easily and quickly cleaned by suction after unravelling the stent. The bronchial lumen was open for ventilation without any occurrence of atelectasis (CT images not shown). Moderate mucus retention was observed proximal to and inside the stent lumen. As expected, we observed granulation tissue, which was most prominent in the proximal and distal ends of the stents.

Day 18: We observed the same phenomenon in the residual distal stent with occurrence of mucus retention and granulation tissue. At this time, the proximal main bronchus from where the proximal stent had been removed was almost free from mucosal inflammation and granulation tissue. The bronchial wall had recovered almost completely 4 days after removal of the stent (Fig. 5 – Day 18).

## Discussion

The new removable SEMS was successfully deployed and accurately placed in the airways; it expanded in accordance with its purpose and remained in a fixed position, both in peripheral and more proximal parts of the right bronchial tree. It was easy to unravel and completely remove without significant medical or technical complications, regarding both the acute and chronic study. The stent was removed carefully as one single metal thread in one sequence. In this ‘proof-of-principle study’, the stent fulfilled its expected efficacy, safety and feasibility. No other studies with predesigned unravelling, removable and uncovered SEMS are known to date. Inflammatory adverse events and granulation tissue, associated with present available uncovered SEMS, also occurred using the new removable stent. To our experience, this phenomenon was shown to be of the same quality and extent as we have seen with ordinary uncovered stents. We emphasise that after the stent removal, the inflammation and granulation tissue had almost disappeared within 4 days, leaving the bronchial wall in a good recovery state.

The current feasibility study was a preclinical proof-of-principle study in presumptively healthy pigs. The results should therefore be interpreted with caution before extended to the treatment of MAO in human beings. The relevance of the animal model for the present hypothesis may therefore be questioned. With respect to the difference between healthy airways of pigs versus MAO in human beings, the findings cannot directly be transferred. Apart from that, the choice of species can be fairly well argued for the main purpose of this initial feasibility study. First, porcine models have been established as reliable animal models for a variety of human lung-related conditions, regarding anatomy and size to genetic and translational medicine, including our own experience (22–24). Testing technical feasibility like deployment and removal of the stent, in both the acute and chronic study, should be very well fitted using this model. Second, assessing the local inflammatory response to the stent material, we emphasise that the porcine model has shown to be the best animal model in the study of wound healing, comparing mammals and humans. The two species have to a large degree similar genome, genetic activation and cellular and subcellular expression of healing processes, including haemostasis and inflammatory responses (25–27). This has been shown to be valid in terms of quality (physiological and pathophysiological process), quantity (degree of reaction) and time perspective (time from trauma or intervention to response), and it is of course partially dependent on study conditions. This study demonstrated an inflammatory airway response similar to the response known in human beings, using ordinary airway SEMS. However, we observed an almost complete recovery of the inflammatory responses 4 days after removal, which supports the importance of not leaving a stent behind for longer than necessary. Third, based on the aspects mentioned above, we find the animal study relevant for the very first feasibility evaluation of the new airway stent, being aware of the limitations mentioned.

Removal of ordinary uncovered SEMS may cause harm to the bronchus and promote bleeding, especially 6–8 weeks after deployment. Covered SEMS can be removed, but also these stents may cause complications due to inflammation, granulation tissue and ingrowth – especially at the proximal and distal ends. In this study, a superficial bleeding occurred after unravelling the stent in the chronic study, which was easily and quickly cleaned by suction and ceased.

To sum up, the pros are 1) easy and accurate deployment with the possibility to change stents during the procedure, 2) stents remained in fixed positions in the airways over 18 days (endoscopy and CT documentation, the latter not shown), 3) easy unravelling and complete removal of the stent without significant technical or medical complications and 4) pronounced and fast mucosal recovery after removal of the stent. Cons are 1) the occurrence of some mucosal retention and 2) inflammatory granulation tissue, as expected in all commercially available stents to date. The new stent functioned according to its purpose and it seems promising for human use.

Area of utilisation may be wide and appropriate. First, uncovered SEMS may maintain airway branch ventilation better than covered or silicon stents, and the new stent may cover all established indications of ordinary SEMS. Second, removable stents may be favourable as short-term or temporary use after interventional bronchoscopy, subsequent extra thoracic radiation or brachytherapy and thereby minimise development of stenosis formation and complicating atelectasis or obstructive pneumonia. The removal of the stent has the potential to further reduce the degree of granulation tissue, thereby achieving an even larger bronchus diameter. Third, the physician would appreciate the opportunity to remove the stent at any time, also during the deployment to guarantee optimal individualised treatment.

There may be some additional advantages: one can order the right size, shape and radial force and vary the thread dimensions and characteristics, and knitting characters. The final stent placement and function has a high potential to be optimal because the stent can be changed at any time. In summary, it may lead to an improved individualised endoscopic therapy. Results from human studies are necessary for final conclusions, but the results from the present proof-of-principle study seem promising for human use.

## Conclusion

The new removable and uncovered self-expanding metal stent was successfully deployed in the central airways of pigs, using a flexible bronchoscope and spontaneously breathing animals. The stents remained fixed in their original positions and were easy to unravel and completely remove from the airways after 14 days, without any clinically significant complications. The removal imposed an almost complete recovery of the inflammatory responses within 4 days. Further studies on animals and humans are needed to evaluate the efficacy, safety and optimal use of the new removable stent in human beings.
